# Chloroplast nucleoids as a transformable network revealed by live imaging with a microfluidic device

**DOI:** 10.1038/s42003-018-0055-1

**Published:** 2018-05-17

**Authors:** Yoshitaka Kamimura, Hitomi Tanaka, Yusuke Kobayashi, Toshiharu Shikanai, Yoshiki Nishimura

**Affiliations:** 1 Department of Botany, Laboratory of Plant Molecular Genetics, Kyoto University, Oiwake-cho, Kita-shirakawa, Sakyo-ku, Kyoto 606-8502 Japan; 2 Department of Cell Genetics, National Institute of Genetics, 1111 Yata, Mishima, Shizuoka, 411-8540 Japan

## Abstract

Chloroplast DNA is organized into DNA–protein conglomerates called chloroplast nucleoids, which are replicated, transcribed, and inherited. We applied live-imaging technology with a microfluidic device to examine the nature of chloroplast nucleoids in *Chlamydomonas reinhardtii*. We observed the dynamic and reversible dispersion of globular chloroplast nucleoids into a network structure in dividing chloroplasts. In the monokaryotic chloroplast (*moc*) mutant, in which chloroplast nucleoids are unequally distributed following chloroplast division due to a defect in *MOC1*, the early stages of chloroplast nucleoid formation occurred mainly in the proximal area. This suggests the chloroplast nucleoid transformable network consists of a highly compact core with proximal areas associated with cpDNA replication and nucleoid formation.

## Abstract (web summary)

Yoshitaka Kamimura and colleagues combine live-imaging technology with microfluidics to examine chloroplast DNA organization in nucleoids. They find that these structures form a network structure in dividing chloroplasts, and propose a mechanism for their inheritance in organelle replication.

## Introduction

Chloroplasts, which are tiny organelles (~5–10 µm) present in plant cells, are involved in the photosynthetic activities responsible for the worldwide annual biomass production of about 100 billion tons^1^. Chloroplasts are presumed to have emerged when a cyanobacterium was engulfed by a non-photosynthetic eukaryotic ancestor cell about 1.2 billion years ago^2^. Chloroplast biogenesis in plants requires the chloroplast genome that was inherited from ancestral bacterial species. The chloroplast genome decreased in size during evolution, but it retained genes indispensable for photosynthesis and the biogenesis of chloroplasts/plastids^3^.

Extant chloroplasts generally possessed ~100 genome copies, with the bulky chloroplast DNA (cpDNA) molecules organized into cpDNA-RNA-protein complexes (i.e., chloroplast nucleoids)^4,5^. Chloroplast nucleoids are thought to be the functional unit for various processes, including DNA replication, repair, recombination, inheritance, and transcription, and are often compared with nuclear chromosomes or bacterial nucleoids^4,5^. Extensive mass spectrometry-based analyses detected multiple nucleoid-associated proteins with multifaceted functions, including core structural proteins as well as proteins related to replication/DNA inheritance, transcription, RNA maturation, translation, and membrane scaffolding^6,7^.

Despite their complex multimeric form, chloroplast nucleoids are not static structures. Their shape, abundance, and distribution changes during the life cycle, differentiation, and evolution of algae and green plants^4,5^. The dynamic shaping of nuclear chromatin and bacterial nucleoids is known to have profound effects on gene expression, inheritance, and other various molecular events. Whereas the mechanisms underlying chromatin remodeling in the nucleus or have been investigated extensively, our understanding on the dynamism of chloroplast nucleoids is still in its infancy^8^. Notably, during chloroplast division, nucleoids appear scattered throughout chloroplasts in green algae^9,10^ and land plants^11^, likely to ensure the faithful and equal inheritance of chloroplast DNA molecules by daughter chloroplasts^10^. However, the actual behaviors of chloroplast nucleoids in dividing chloroplasts remain unknown. In this report, we aimed to capture the live movements of chloroplast nucleoids during chloroplast division to elucidate their behaviors and structural nature, which revealed that cp nucleoids are interconnected transformable network consisting of a highly compact core and peripheral/interconnecting areas associated with replication hot spots.

## Results

### Establishing monitoring system for chloroplast nucleoids

**Fig. 1:**
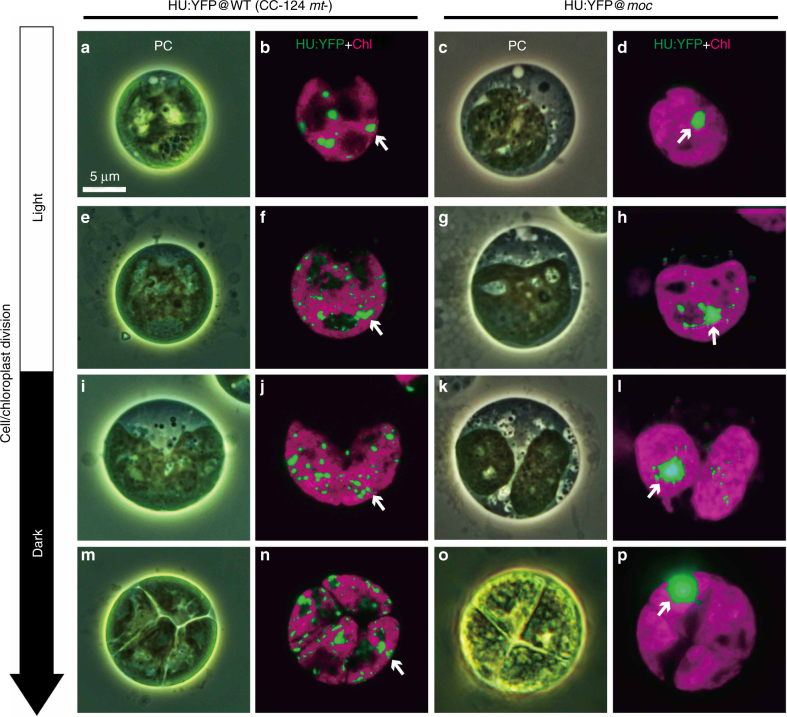
Chloroplast nucleoid behaviors in the dividing cells and chloroplasts of wild-type (CC-124 *mt-*) and *moc* mutant samples. Chloroplast nucleoid behaviors (arrows) in dividing cells and chloroplasts were monitored in the wild-type control (WT; CC-124 *mt-*; left panel: **a**, **b**, **e**, **f**, **i**, **j**, **m**, and **n**) and *moc* mutant (right panel: **c**, **d**, **g**, **h**, **k**, **l**, **o**, and **p**). Chloroplast nucleoids were labeled with HU:YFP. During the G_1_ phase of the WT control (**a**, **b**), chloroplast nucleoids were observed as 5–10 yellow spots, whereas they aggregated into one nucleoid in the *moc* mutant (**c**, **d**). Upon the initiation of cell/chloroplast division, chloroplast nucleoids were scattered in WT cells (**e**, **f**, **i**, and **j**), but less so in the *moc* mutant (**g**, **h**, **k**, and **l**). Chloroplast nucleoids were eventually equally distributed in four daughter chloroplasts in WT cells (**m**, **n**), but in the *moc* mutant, the aggregated chloroplast nucleoid was transmitted to only one daughter chloroplast. The remaining three daughter chloroplasts did not receive visible chloroplast nucleoids (**o**, **p**)

The unicellular green alga *Chlamydomonas reinhardtii* is an ideal model organism for the live imaging of chloroplast nucleoids because each of its cells harbors one chloroplast and the divisions can be readily synchronized by controlling the light/dark condition. Additionally, ~80 cpDNA copies are organized into 5–10 chloroplast nucleoids (~1.5 µm) that can be clearly observed by fluorescence microscopy (Fig. 1).

However, the following two issues must be resolved before chloroplast nucleoid movements in living *C. reinhardtii* cells can be monitored: chloroplast nucleoids should be labeled specifically and motile algae must be trapped to be observed microscopically. Although SYBR Green I is a commonly used fluorochrome for visualizing DNA molecules in living cells^12^, it is unstable and unsuitable for time-lapse imaging. Furthermore, in addition to chloroplast nucleoids, SYBR Green I stains mitochondrial nucleoids and cell nuclei, making it difficult to precisely track chloroplast nucleoid movements. Therefore, we prepared a strain expressing a yellow fluorescent protein (YFP) fused to the heat unstable (HU) protein, which is one of the major chloroplast nucleoid proteins^7,13^. The fluorescence of HU:YFP was stable throughout the cell cycle. Additionally, the morphological changes to chloroplast nucleoids were clearly visualized during cell/chloroplast divisions, which were almost discernible in the images of SYBR Green I-stained cells^9,10^ (Fig. 1). A microfluidic system was employed to trap viable cells under the microscope over a prolonged period (>12 h) (Supplementary Fig. 1). The microfluidic system comprised a micro-chamber and pumps. The *C. reinhardtii* cells (5–10 µm) were infused into the chamber where they were trapped between narrow gaps (3.5–4.5 µm). The cells were continuously provided fresh growth medium (Tris–acetate–phosphate: TAP) to maintain their viability. This microfluidic platform was placed on the stage of a confocal laser microscope and *Z*-stack images were obtained periodically. This system eventually visualized chloroplast nucleoid behaviors during chloroplast division in living cells (Supplemental Movie 1).

### Dynamic transformation of chloroplast nucleoids into network

**Fig. 2:**
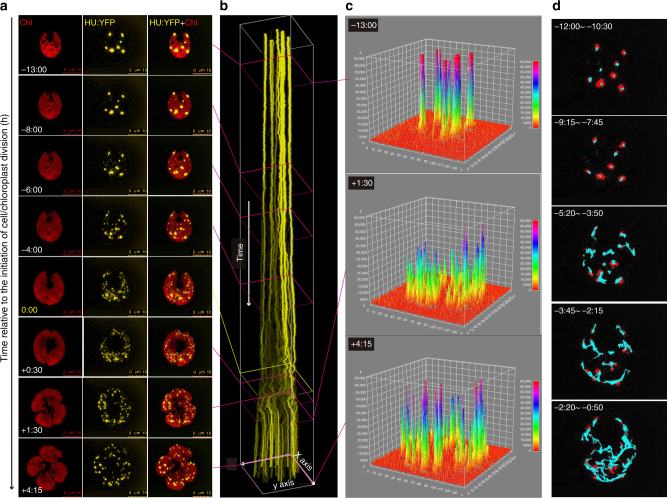
Reversible transformation of particulate chloroplast nucleoids into a network during cell/chloroplast division. Time-lapse images of a *Chlamydomonas reinhardtii* cell in a microfluidic device. Chlorophyll fluorescence (Chl), YFP fused to HU (HU:YFP), and merged (HU:YFP+Chl) images are presented. The times relative to the initiation of cell/chloroplast division are indicated in the lower left corner of each Chl panel (**a**). Kymographs representing chloroplast nucleoid division and fusion. Magenta squares represent the time points corresponding to the panels in (**a**) and (**c**). The yellow square corresponds to the initiation of cell/chloroplast division (0:00) (**b**). Surface plot analysis revealing changes in HU:YFP signal intensity and distribution (**c**). At −13:00, peaks with high fluorescence intensity (>65,000 arbitrary unit (a.u.)) were observed. The relative fluorescence intensity of the peaks gradually decreased and new peaks with lower fluorescence were observed until +1:30 (i.e., immediately before cell/chloroplast division). The number of peaks decreased and the fluorescence intensity of each peak recovered to ~80% (at +4:15) after the cells and chloroplasts finished dividing. This reflected the fusion of the chloroplast nucleoids and the replication of cpDNA (**c**). Particle-tracking analysis (**d**). The movements of HU:YFP signals over 1.5 h are indicated (blue lines). The HU:YFP signals (chloroplast nucleoids) at the end of the tracking period are presented in red

During the G_1_ phase, 5–10 chloroplast nucleoids (yellow) were observed in one chloroplast (Fig. 2a; Supplemental Movie 2). The chloroplast nucleoids were globular (~1.5 µm) and occurred independently. However, at about 6 h before the initiation of cell/chloroplast division (−6:00), small particles (~0.1–0.5 µm) started to detach from the chloroplast nucleoids, which decreased in size as the number of particles increased. Additionally, the small particles moved back and forth to connect the chloroplast nucleoids. Eventually, chloroplast nucleoids formed a network-like structure that spread throughout the chloroplast, which divided twice to form four daughter chloroplasts after the network-like structure formed. The small particles quickly fused to form a separate globular structure after the chloroplast divided (Fig. 2a; Supplemental Movie 2).

The division and fusion of chloroplast nucleoids were clearly visualized in a kymograph, which is a graphical representation of spatial positions over time (Fig. 2b). In the kymograph, each yellow column, which represented a single chloroplast nucleoid, remained almost unchanged until −6:00, after which considerable branching was observed, indicative of extensive nucleoid divisions. Meanwhile, the fusing of fine branches was observed upon completion of the cell/chloroplast division at +1:30.

This process was further investigated by conducting three-dimensional surface plot analyses (Fig. 2c). At −13:00, peaks with high fluorescence intensity (>65,000 arbitrary unit (a.u.)) were observed, and they remained unchanged until −6:00. The relative fluorescence intensity of the peaks gradually decreased, while new peaks with lower fluorescence (25,000–35,000 a.u.) emerged until +1:30 (i.e., immediately before cell/chloroplast division). The number of peaks decreased after the cells and chloroplasts divided, and the fluorescence intensity of each peak recovered to ~80% (at +4:15). This reflected the fact that the chloroplast nucleoids had fused and the cpDNA had replicated (Fig. 1). In contrast to the relatively slow (i.e., almost 6 h) dispersal of the chloroplast nucleoids before the cells and chloroplasts divided, the chloroplast nucleoids fused within 2–3 h.

Chloroplast nucleoid movement was examined by particle-tracking analysis (Fig. 2d). Particle movements were recorded at various time points over 1.5 h, after which the detected movements were compared. All of the chloroplast nucleoids were almost motionless from −12:00 to −8:00, with only subtle tremors detected. At ~−6:00, minor particle movements were observed, mainly in the area connecting chloroplast nucleoids. Increasing chloroplast nucleoid movements were observed starting at −3:00. Movement signals were detected at all locations linking chloroplast nucleoids between −2:20 to 0:50, suggesting the chloroplast nucleoid structure formed a network that spread throughout the chloroplast (Fig. 2d).

These observations imply that the classical view of chloroplast nucleoids, which assumed they are distinct particulate structures isolated from each other, needs to be revised. Specifically, chloroplast nucleoids are likely components of a dynamic network in which several condensed areas are surrounded by a network-like cpDNA–protein structure that is dispersed throughout the chloroplast. Additionally, the condensed areas can be actively disintegrated and reformed in dividing cells and chloroplasts.

### The early process of chloroplast nucleoid formation

A monokaryotic chloroplast (*moc*) mutant was analyzed to further clarify chloroplast nucleoid behaviors. This mutant possesses only a single chloroplast nucleoid and shows unequal segregation of chloroplast nucleoids during chloroplast divisions (Fig. 1), due to a defect in the gene *MOC1* encoding Holliday junction resolvase (HJR). The HJR enzyme introduces symmetrical endonucleolytic cleavages at the core of Holliday junctions in a sequence- and structure-dependent manner, which is critical for disentangling chloroplast DNA molecules in dividing chloroplasts. An earlier study revealed that *MOC1* gene, which is required for the final step of homologous recombination, is conserved from green algae to land plants. Reduced or no expression of *MOC1* gene lead to aberrant chloroplast nucleoid aggregation and growth defects in the land plant *Arabidopsis thaliana*^10^.

Chloroplast nucleoids in the *moc* mutant and wild-type (WT) cells were similarly labeled with HU:YFP (Fig. 1). Aggregated chloroplast nucleoids in the G_1_ phase were clearly visualized. There was no clear indication that the chloroplast nucleoids disintegrated into a network-like structure following cell and chloroplast division in the *moc* mutant. Moreover, the aggregated chloroplast nucleoids were mostly inherited by one of the four daughter cells, leaving the remaining daughter cells apparently devoid of chloroplast nucleoids. These results imply that the HJR encoded by *MOC1* is critical for regulating the dispersal of chloroplast nucleoids. Furthermore, this active dispersal is probably required for the stochastic equal distribution of chloroplast nucleoids to the daughter cells (Fig. 1).

Curiously, the results of a previous study^14^ indicated the three daughter cells that fail to inherit the aggregated chloroplast nucleoids do not die, and there are almost no differences in the growth rates of these and WT cells. These mutant cells may survive by rapidly replicating cpDNA to levels comparable to those of the parental cells. Thus, we focused on the behaviors of *moc*-type chloroplast nucleoids after the cells and chloroplasts divided (Supplemental Movies 3 and 4).

**Fig. 3:**
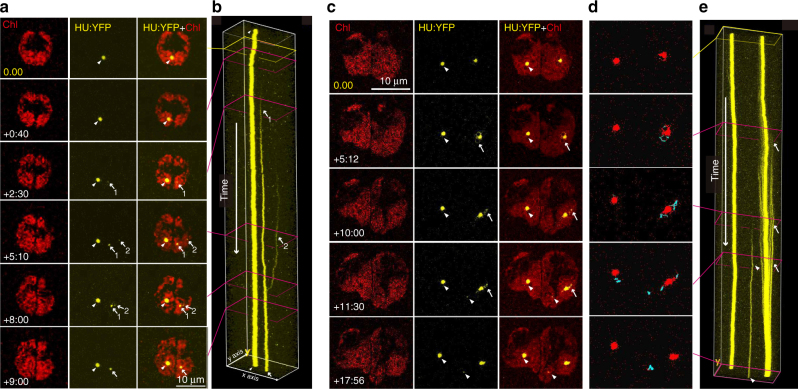
De novo emergence and replication of chloroplast nucleoids in the *moc* mutant. Time-lapse images of a *moc* cell in a microfluidic device. Chlorophyll fluorescence (Chl), YFP fused to HU (HU:YFP), and merged (HU:YFP+Chl) images are shown. The times relative to the initiation of cell/chloroplast division are indicated in the lower left corner of each Chl panel. Arrowheads indicate pre-existing *moc*-type chloroplast nucleoids preferentially inherited by one chloroplast (left). White arrows indicate the de novo emergence of two minute chloroplast nucleoids (1 and 2) in the chloroplast that failed to inherit the *moc-*type chloroplast nucleoid (right) (**a**). Kymographs representing the emergence of two chloroplast nucleoids (arrows 1 and 2) and the fusion of chloroplast nucleoids (**b**). Magenta squares represent the time points corresponding to the panels in (**a**). The yellow square indicates the initiation of cell/chloroplast division (0:00) (**b**). Time-lapse images of a *moc* cell revealing the replication of chloroplast nucleoids (**c**). Arrowheads indicate pre-existing *moc*-type chloroplast nucleoids. Arrows indicate the loops and minor particles that emerged around the new chloroplast nucleoid. Particle-tracking analysis during the replication of a new chloroplast nucleoid (**d**). The movements of HU:YFP signals over 1.5 h are indicated (blue lines). The HU:YFP signals (chloroplast nucleoids) at the end of the particle-tracking period are presented in red. The blue signals were preferentially detected around the new chloroplast nucleoid (right) (**d**). A kymograph revealed the movements of small particles, which formed a comet tail-like pattern, indicative of the emergence and fusion of numerous chloroplast nucleoid particles (arrows) (**e**)

Dividing chloroplast nucleoids were not detected during cell/chloroplast division, confirming the importance of cpHJR in the active dispersal of chloroplast nucleoids (Fig. 3; Supplemental Movie 3). However, at 2.5 h after cells and chloroplasts had divided (+2:30), a minute chloroplast nucleoid was observed emerging from the apparently empty space within the chloroplast. A second chloroplast nucleoid emerged at +5:10. These two newly emerged chloroplast nucleoids slowly approached each other and eventually merged. The emergence and fusion of the two new minor chloroplast nucleoids were clearly visualized in the kymograph. These observations indicate that even in the absence of obviously dividing chloroplast nucleoids, an undetectable amount of cpDNA molecules may be inherited by daughter cells to be used as templates for de novo chloroplast nucleoid formation (Fig. 3; Supplemental Movie 3).

Another movie of *moc*-type chloroplast nucleoids revealed what happens to newly formed chloroplast nucleoids in growing cells (Fig. 3; Supplemental Movie 4). In this movie, the cell and chloroplast had divided before we started recording. Consequently, the starting point was arbitrarily set as 0:00. At +5:12, loop-like structures emerged and flickered in the area surrounding the minor chloroplast nucleoid. The small particles that comprised the loops were gradually absorbed into the minor chloroplast nucleoid. Another group of particles then emerged and fluttered before being gradually absorbed into the minor chloroplast nucleoid core. This process was observed until +11:30, and the minor chloroplast nucleoid grew until it was almost the same as the major chloroplast nucleoid in terms of size (Fig. 3; Supplemental Movie 4).

The kymograph revealed a comet tail-like structure, which corresponded to the repeated emergence and fusion of chloroplast nucleoid particles (Fig. 3). These results suggest that cpDNA synthesis and chloroplast nucleoid formation do not occur at the center of established chloroplast nucleoids, but instead occur at the proximal areas, surrounding the core of chloroplast nucleoids. The particle-tracking analysis indicated that this event is specific to the minor chloroplast nucleoid, suggesting that there may be mechanisms linking the measurement of chloroplast nucleoid size and the rate of cpDNA synthesis (Fig. 3; Supplemental Movie 5).

### Hot spots for cpDNA synthesis outside of cp nucleoids

**Fig. 4:**
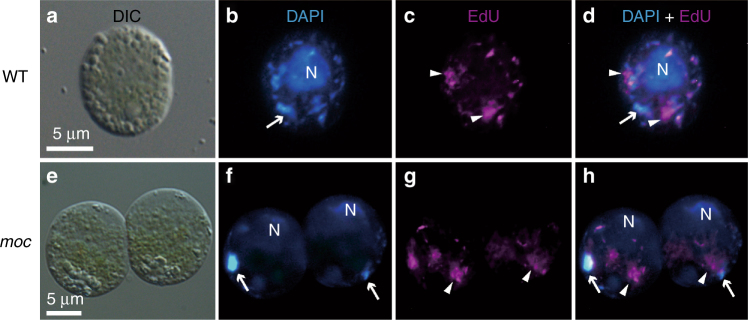
Hot spots for de novo cpDNA synthesis visualized by EdU labeling. Wild-type (WT) (**a**–**d**) and *moc* mutant (*moc*) vegetative cells (**e**–**h**) were labeled with EdU immediately before cells and chloroplasts divided to visualize hot spots for cpDNA synthesis. Differential interference (DIC: **a**, **e**)), DAPI (blue: **b**, **f**), EdU (magenta: **c**, **g**), and merged DAPI and EdU (DAPI + EdU: **d**, **h**) images are presented. Arrows indicate the chloroplast nucleoid positions, while the arrowheads correspond to the hot spots for de novo cpDNA synthesis. N cell nucleus

The sites of de novo cpDNA synthesis were assessed with 5-ethynyl-2′-deoxyuridine (EdU) (Fig. 4), which is a thymidine analog that can be incorporated into newly synthesized DNA molecules^15^. The incorporated EdU can be detected in milder conditions than those required for bromo-deoxyuridine (BrdU) (i.e., DNA denaturation with HCl). The incorporation of EdU into cpDNA is reportedly enhanced by 5-fluoro-2′-deoxyuridine, which depletes the thymidine pool within chloroplasts^16,17^. In this study, we visualized the sites of de novo cpDNA replication at the periphery and outside of DAPI-stained chloroplast nucleoids (Fig. 4). The signal was clearer in *moc* mutants, in which a network of de novo cpDNA synthesis sites were observed. These results indicate that cpDNA at the periphery of the nucleoids and in the interconnected areas are actively replicated, with decreased cpDNA compaction by nucleoid proteins. These results are consistent with those of an earlier study of mitochondrial nucleoids, which concluded the binding of a major nucleoid protein (mitochondrial transcription factor A) to mtDNA inhibits replication in vitro^18^.

## Discussion

Chloroplast nucleoids in most green algae, mosses, and vascular plants are generally assumed to be complex DNA–RNA-protein structures with multiple RNA and protein components^4,19–22^. Our data indicate that chloroplast nucleoids form a dynamic network with several nodes compacted by some core chloroplast nucleoid proteins (Supplementary Fig. 2). The compacted core of chloroplast nucleoids can be dynamically unfolded as required (e.g., during chloroplast division). Moreover, the abundance of cpHJR, which is one of the key factors involved in this unfolding process, increases after chloroplasts divide. The cpHJR enzyme also introduces nicks into cpDNA molecules that enable the segregation of HJs, thereby relieving torsional stress^10^. Our data also showed that the dynamic disintegration and reformation of spherical chloroplast nucleoids are precisely coordinated with the timing of cell/chloroplast division (Fig. 2 and Supplemental Movie 2). Interestingly, similar chloroplast nucleoid behaviors have been observed in *Arabidopsis thaliana*^11^, suggesting the associated mechanisms may be conserved among green plants.

CpDNAs have generally been considered to be circular. However, it is more likely that cpDNA molecules in vivo actually consist of a mixture of monomers and concatemers with complex branched structures driven by a recombination-dependent replication process^19,20^. This view might be consistent with our microscopic observations, which suggests cpDNA and chloroplast nucleoids form a network-like structure.

Our *moc* mutant (Fig. 3) and EdU labeling (Fig. 4) analyses imply that the initial formation of chloroplast nucleoids does not occur at the center of chloroplast nucleoids. Instead, it likely takes place in the proximal area surrounding chloroplast nucleoids where cpDNA molecules are probably more relaxed, allowing newly synthesized chloroplast nucleoid proteins, DNA polymerases, gyrases, and topoisomerases to access the cpDNA with relative ease. Upon the recruitment of HU, gyrases, and topoisomerases, some topological changes, such as bending and torsional stress, are introduced to cpDNA molecules to enable the compaction of bulky cpDNA molecules into spherical structures within the limited stromal area (Supplementary Fig. 2). These processes were likely observed as the emergence, flickering, and absorbance of small fluorescent particles into the chloroplast nucleoid core (Fig. 3 and Supplementary Movie 4).

Early electron microscopy-based studies of bacteria suggested that a nucleoid consists of a central core, from which supercoiled loops emanate^23,24^. The presence of such topologically isolated domains in bacterial nucleoids is also suggested by chromosome conformation capture combined with deep sequencing, or Hi-C^25^. Determining whether similar topological domains can be detected in chloroplast nucleoids is warranted. The transient loops that emerged around *moc-*type chloroplast nucleoids (Fig. 2 or Supplementary Movie 4) may indicate the presence of such dynamic structures in chloroplast nucleoids. The relationship between the morphology of chloroplast nucleoids and transcription/translation should also be clarified. The chloroplast nucleoid core may be compared with the heterochromatic region in the eukaryotic cell nucleus^21^. Interestingly, a previous in situ hybridization analysis suggested that the active spots for translation (translation (T) zone) in chloroplasts may be distinct from chloroplast nucleoids^26^. Future studies should also address whether methylation, which probably enhances cpDNA replication^27,28^, helps regulate the chloroplast nucleoid structure. Moreover, additional research will be required to determine whether morphological changes to chloroplast nucleoids in response to environmental conditions (e.g., availability of phosphate^29^ and light) as well as in different developmental stages are linked to transcriptional regulation. The detailed mechanisms that govern the morphology of chloroplast nucleoids would be illuminated by addressing these issues.

## Methods

### Culture conditions

*C. reinhardtii* cells were cultured in TAP medium on a shaker (120 r.p.m.) at 23 °C. Cells were exposed to a 14-h light (30 µmol photons m^−2^ s^−1^): 10-h dark cycle to synchronize cell division. Cells at the log growth phase were analyzed.

### Microscopic observation

Cells were observed using a BX51 fluorescence/differential interference microscope (Olympus, Tokyo, Japan) connected to a DP72 charge-coupled device camera (Olympus). To stain DNA in living cells, SYBR Green I (Thermo Fisher Scientific, Waltham, MA, USA) was added to the cell suspensions at a dilution of 1:1000. For DAPI (4′,6-diamidino-2-phenylindole) staining, cells were fixed in 3.7% formaldehyde and incubated in 0.5 µg/mL DAPI (Nacalai Tesque, Kyoto, Japan). After adding the fluorochromes, cell suspensions were incubated for 5–10 min at room temperature.

### Live imaging using a microfluidic device

CellASIC™ ONIX Y04C-02 Microfluidic Yeast Plates were used with the CellASIC™ Microfluidic system and CellASIC™ ONIX F84 manifolds (Merck, Kenilworth, NJ, USA) for the live imaging of the chloroplast nucleoids in *C. reinhardtii* cells (Supplementary Fig. 2). Prior to the experiments, TAP medium supplemented with 0.1% ampicillin (TAP + Amp) was added to the inlet well and distilled water was added to the cell inlets. They were perfused into the microfluidic plate at 5 and 8 psi, respectively. The waste in the outlet was removed following the pre-treatment, after which 50 µL cell suspension was added to the cell inlets and 350 µL TAP + Amp was added to the inlet well. The plate was sealed tightly with the manifold and placed on the stage of the SP5 confocal scanning laser microscope (Leica Microsystems, Wetzlar, Germany) equipped with one Hybrid detector (HyD) and two photon multiplier tubes (PMTs). Cells were loaded into the micro-chamber by applying a pressure (8 psi) to cell inlets for 5–8 s. The number, distribution, and condition of cells were checked microscopically and the loading process was repeated as necessary. The TAP + Amp medium was provided continuously to the chamber throughout the experiment with a low pressure (1 psi) at the inlet well. Argon laser (output 20%, laser power 0.1%) was used for the time-lapse monitoring to minimize photodamage to the cells. HyD and PMT1 was used to detect YFP signal (520–582.3 nm) and chlorophyll signal (661–721 nm), respectively.

### Image analysis

Images were analyzed with the ImageJ software (National Institutes of Health, USA). Kymographs were generated using the Reslice function. A surface plot was used to monitor the distribution and intensity of the HU:YFP fluorescence. The particle-tracking analysis was conducted with the Particle Track and Analysis plugin (http://www.sanken.osaka-u.ac.jp/labs/bse/ImageJcontents/frameImageJ-en.html).

### Imaging of cpDNA synthesis with EdU

The Click-iT EdU imaging kit with Alexa Fluor 488 (Invitrogen, Carlsbad, CA, USA) was used for EdU (5-ethynyl-2′-deoxyuridine) imaging analysis. We added 1 mM EdU to the suspensions of synchronized cells 1 h before the dark period. To enhance the incorporation of EdU to cpDNA, the thymidine pool in chloroplasts was depleted by adding 1 mM FdUrd (5-fluoro-2′-deoxyuridine)^16,17^. After a 4-h incubation, the cells were fixed with 3.7% formaldehyde for 10 min on ice. Cells were collected by a centrifugation at 3000 r.p.m. for 1 min, washed twice with ice-cold wash buffer (3% bovine serum albumin and 10 mM HEPES (pH 6.8)), and then permeabilized with a buffer containing 5% Triton, 3% bovine serum albumin, and 10 mM HEPES (pH 6.8) for 20 min on ice. After washing twice with ice-cold wash buffer, the precipitated cells were suspended in the Click-iT reaction mix and incubated for 30 min on ice. Samples were not incubated at room temperature to avoid the non-specific staining of oil bodies. Cells were washed twice with the wash buffer, co-stained with DAPI, and observed with the BX51 microscope.

### Data availability

The authors declare that all data supporting the findings of this study are available within the article and its supplementary information files. The plasmids (pNYAN and HU@pNYAN) and strains (CC-124 (WT) *mt-*, *moc1 mt-*, with or without the introduction of HU@pNYAN) used in this study are available upon request. The sequence of plasmids are available in Supplementary Note 1.

## Electronic supplementary material

Supplementary Information

Description of Additional Supplementary Files

Supplemental Movie 1

Supplemental Movie 2

Supplemental Movie 3

Supplemental Movie 4

Supplemental Movie 5
