# Supplementary material for: Chloroplast nucleoids as a transformable network revealed by live imaging with a microfluidic device
Source: Commun Biol. 2018 May 17;1:47. doi: 10.1038/s42003-018-0055-1 (PMC6123815; doi:10.1038/s42003-018-0055-1)
Supplement: Supplementary file 1 — Supplementary Information [file 42003_2018_55_MOESM1_ESM.pdf]

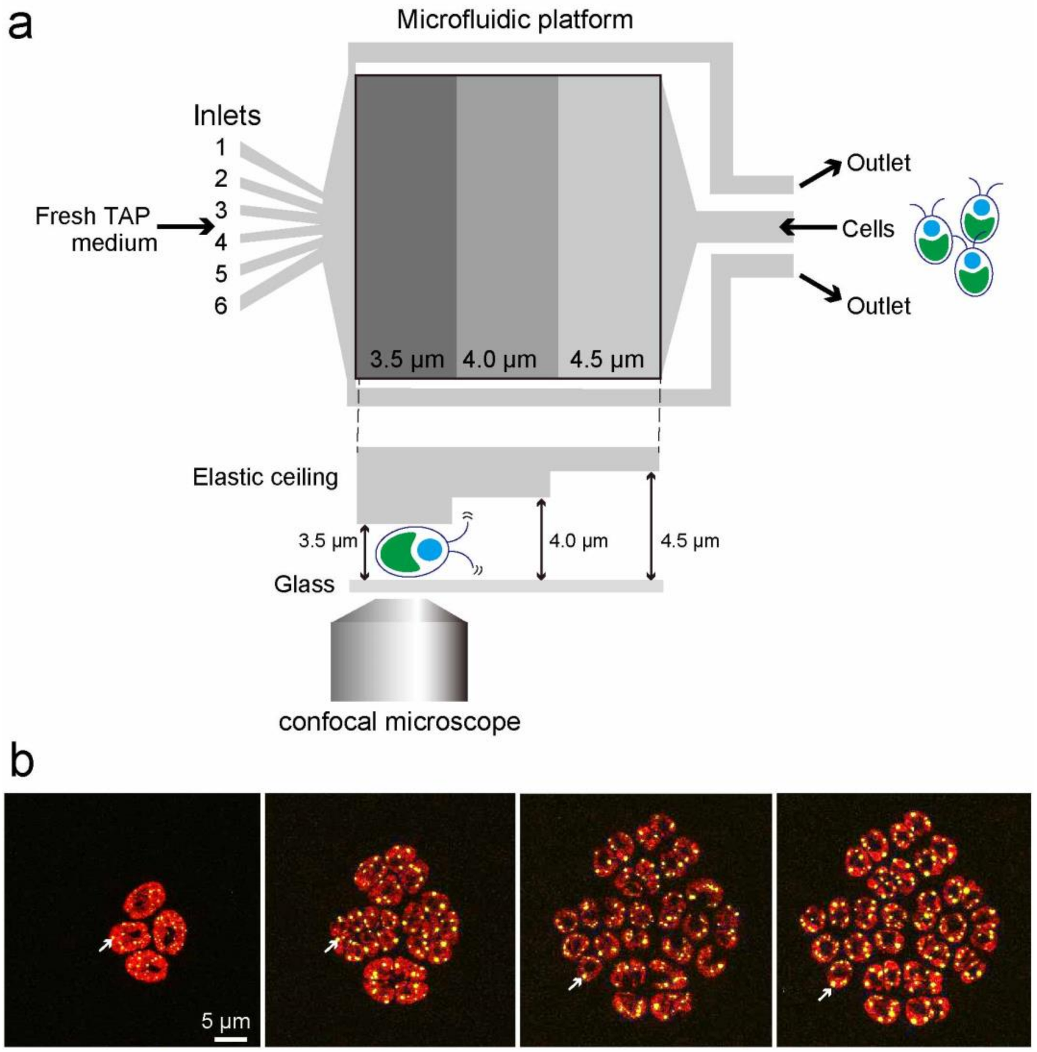

Supplementary Figure 1. Live-imaging of dividing chloroplast nucleoids with a microfluidic device.

(a) Schematic drawing of the microfluidic device employed to visualize the chloroplast nucleoid behaviors in dividing cells and chloroplasts. *Chlamydomonas reinhardtii* cells were trapped between 3.5–4.5 µm gaps in the micro-chamber. The trapped cells were kept alive by continuously providing fresh TAP medium to the platform. Time-lapse Z-stack images were collected using a confocal microscope. (b) Live images of dividing and proliferating *C. reinhardtii* cells expressing HU:YFP. Chloroplast nucleoids were visualized as yellow particles (arrows). Autofluorescence emitted from the chlorophyll in chloroplasts is indicated in red.

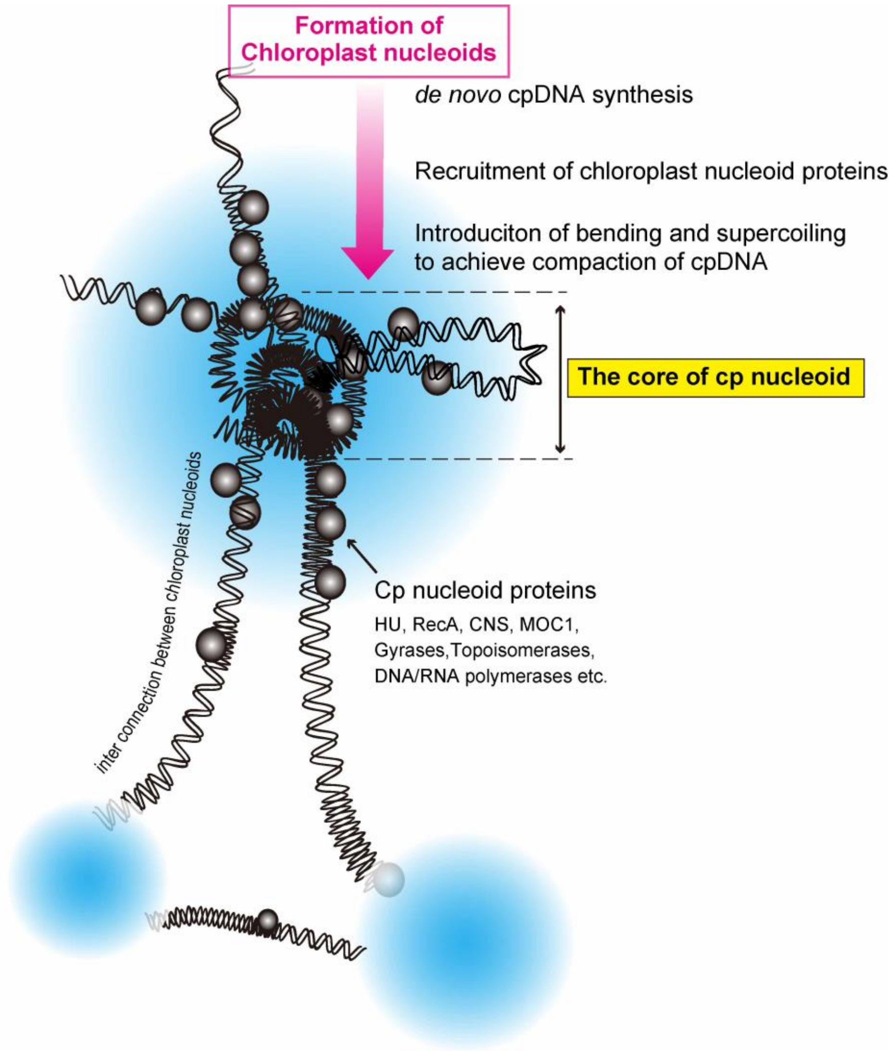

Supplementary Figure 2. Schematic model of the transformable chloroplast nucleoid network structure.

Chloroplast nucleoids have been described as distinct particles in green plants (represented by blue clouds), but our analyses suggest that they are interconnected by an undetectable amount of fine cpDNA–protein fibers. Chloroplast nucleoids start to form mainly at the periphery of pre-existing chloroplast nucleoids where cpDNA molecules might be relatively relaxed. Various chloroplast nucleoid proteins are subsequently recruited to the *de novo* synthesized cpDNA, causing the cpDNA molecules to bend and twist. The resulting increase in torsional stress causes the cpDNA molecules to form supercoils and plectoneme-like structures. This leads to the compaction and eventual fusion of newly formed chloroplast nucleoids. Additionally, critical triggers for

the disintegration of particulate chloroplast nucleoids upon cell/chloroplast division involve the dissociation of entangled cpDNA molecules by MOC1 (Holliday junction resolvase). The concomitant relaxation of torsional stresses within cpDNA molecules eliminates supercoils and plectonemes, leading to the dispersal of cpDNA molecules and the eventual disintegration of particulate structures into a network.

Supplementary Note 1. Sequences of plasmids pNYAN and HU@pNYAN.

>pNYAN

GAGCTCCACCGCGGTGGCGGCCGCTCTAGCTAGAACTAGTGGATCGATCCCACACACC
TGCCCGTCTGCCTGACAGGAAGTGAACGCATGTCGAGGGAGGCCTCACCAATCGTCACA
CGAGCCCTCGTCAGAAACACGTCTCCGCCACGCTCTCCCTCTCACGGCCGACCCCGCAG
CCCTTTTGCCCTTTCCTAGGCCACCGACAGGACCCAGGCGCTCTCAGCATGCCTCAACA
ACCCGTACTCGTGCCAGCGGTGCCCTTGTGCTGGTGATCGCTTGGAAGCGCATGCGAAG
ACGAAGGGGCGGAGCAGGCGGCCTGGCTGTTCGAAGGGCTCGCCGCCAGTTCGGGTGCC
TTTCTCCACGCGCGCCTCCACACCTACCGATGCGTGAAGGCAGGCAAATGCTCATGTTT
GCCCGAACTCGGAGTCCTTAAAAAGCCGCTTCTTGTCGTCGTTCCGAGACATGTTAGCA
GATCGCAGTGCCACCTTTCCTGACGCGCTCGGCCCCATATTCGGACGCAATTGTCATTT
GTAGCACAATTGGAGCAAATCTGGCGAGGCAGTAGGCTTTTAAGTTGCAAGGCGAGAGA
GCAAAGTGGGACGCGGCGTGATTATTGGTATTTACGCGACGGCCCGGCGCGTTAGCGGC
CCTTCCCCCAGGCCAGGGACGATTATGTATCAATATTGTTGCGTTCGGGCACTCGTGCG
AGGGCTCCTGCGGGCTGGGGAGGGGGATCTGGGAATTGGAGGTACGACCGAGATGGCTT
GCTCGGGGGGAGGTTTCCTCGCCGAGCAAGCCAGGGTTAGGTGTTGCGCTCTTGACTCG
TTGTGCATTCTAGGACCCCACTGCTACTCACAACAAGCCCATATGGAATTCGGCGGCT
CCGGCGGCATGGTGAGCAAGGGCGAGGAGCTGTTCACCGGGGTGGTGCCCATCCTGGTC
GAGCTGGACGGCGACGTAAACGGCCACAAGTTCAGCGTGTCCGGCGAGGGCGAGGGCGA
TGCCACCTACGGCAAGCTGACCCTGAAGCTGATCTGCACCACCGGCAAGCTGCCCGTGC
CCTGGCCCACCCTCGTGACCACCCTGGGCTACGGCCTGCAGTGCTTCGCCCGCTACCCC
GACCACATGAAGCAGCACGACTTCTTCAAGTCCGCCATGCCCGAAGGCTACGTCCAGGA
GCGCACCATCTTCTTCAAGGACGACGGCAACTACAAGACCCGCGCCGAGGTGAAGTTCG
AGGGCGACACCCTGGTGAACCGCATCGAGCTGAAGGGCATCGACTTCAAGGAGGACGGC
AACATCCTGGGGCACAAGCTGGAGTACAACTACAACAGCCACAACGTCTATATCACCGC
CGACAAGCAGAAGAACGGCATCAAGGCCAACTTCAAGATCCGCCACAACATCGAGGACG
GCGGCGTGCAGCTCGCCGACCACTACCAGCAGAACACCCCCATCGGCGACGGCCCCGTG
CTGCTGCCCGACAACCACTACCTGAGCTACCAGTCCGCCCTGAGCAAAGACCCCAACGA
GAAGCGCGATCACATGGTCCTGCTGGAGTTCGTGACCGCCGCCGGGATCACTCTCGGCA
TGGACGAGCTGTACAAGTAACGATACCCCTACGACGTGCCCGACTACGCCTACCCCTAC
GACGTGCCCGACTACGCCGATCGATCCGGACCGTACCCCTACGACGTGCCCGACTACGC

CGCTAGCAGTATCTAACTGAGTAATTCTGGCAGCAGCTGGACCGCCTGTACCATGGAGA
AGAGCTTTACTTGCCGGGATGGCCGATTTCGCTGATTGATACGGGATCGGAGCTCGGAG
GCTTTCGCGCTAGGGGCTAGGCGAAGGGCAGTGGTGACCAGGGTCGGTGTGGGGTCGGC
CCACGGTCAATTAGCCACAGGAGGATCAGGGGGAGGTAGGCACGTCGACTTGGTTTGCG
ACCCCGCAGTTTTGGCGGACGTGCTGTTGTAGATGTTAGCGTGTGCGTGAGCCAGTGGC
CAACGTGCCACACCCATTGAGAAGACCAACCAACTTACTGGCAATATCTGCCAATGCCA
TACTGCATGTAATGGCCAGGCCATGTGAGAGTTTGCCGTGCCTGCGCGCGCCCCGGGGG
CGGGGGGGGGACGGGTGGGGGGTAGGGGGTCTCACGGGAACAGCACGCTAGGGGTCAGG
GGGGGGGGGGGCGCAGTTTAGCTGACCAGCCGTGGGATGATGCACGCATTTGCAAGGA
CAGGGTAATCACAGCAGCAACATGGTGGGCTTAGGACAGCTGTGGGTCAGTGGACGGAC
GGCAGGGGAGGGACGGCGCAGCTCGGGAGACAGGGGGAGACAGCGTGACTGTGCAATCA
AGCTTATCGATACCGTCGACCTCGACGCTGAGGCTTGACATGATTGGTGCGTATGTTTG
TATGAAGCTACAGGACTGATTTGGCGGGCTATGAGGGCGGGGGAAGCTCTGGAAGGGCC
GCGATGGGGCGCGCGGCGTCCAGAAGGCGCCATACGGCCCGCTGGCGGCACCCATCCGG
TATAAAAGCCCGCGACCCCGAACGGTGACCTCCACTTTCAGCGACAAACGAGCACTTAT
ACATACGCGACTATTCTGCCGCTATACATAACCACTCAGCTAGCTTAAGATCCCATCAA
GCTTGCATGCCGGGCGCGCCAGAAGGAGCGCAGCCAAACCAGGATGATGTTTGATGGGG
TATTTGAGCACTTGCAACCCTTATCCGGAAGCCCCCTGGCCCACAAAGGCTAGGCGCCA
ATGCAAGCAGTTCGCATGCAGCCCCTGGAGCGGTGCCCTCCTGATAAACCGGCCAGGGG
GCCTATGTTCTTTACTTTTTTACAAGAGAAGTCACTCAACATCTTAAAATGGCCAGgtg
agtcgacgagcaagcccggcggatcaggcagcgtgcttgcagatttgacttgcaacgcc
cgcattgtgtcgacgaaggcttttggctcctctgtcgctgtctcaagcagcatctaacc
ctgcgtcgccgtttccatttgcagGATGGCCACTCCGCCCTCCCCGGTGCTGAAGAATT
TCGAAGCATGGACGATGCGTTGCGTGCACTGCGGGGTCGGTATCCCGGTTGTGAGTGGG
TTGTTGTGGAGGATGGGGCCTCGGGGGCTGGTGTTTATCGGCTTCGGGGTGGTGGGCGG
GAGTTGTTTGTCAAGGTGGCAGCTCTGGGGGCCGGGGTGGGCTTGTTGGGTGAGGCTGA
GCGGCTGGTGTGGTTGGCGGAGGTGGGGATTCCCGTACCTCGTGTTGTGGAGGGTGGTG
GGGACGAGAGGGTCGCCTGGTTGGTCACCGAAGCGGTTCCGGGGCGTCCGGCCAGTGCG
CGGTGGCCGCGGGAGCAGCGGCTGGACGTGGCGGTGGCGCTCGCGGGGCTCGCTCGTTC
GCTGCACGCGCTGGACTGGGAGCGGTGTCCGTTCGATCGCAGTCTCGCGGTGACGGTGC
CGCAGGCGGCCCGTGCTGTCGCTGAAGGGAGCGTCGACTTGGAGGATCTGGACGAGGAG
CGGAAGGGGTGGTCGGGGGAGCGGCTTCTCGCCGAGCTGGAGCGGACTCGGCCTGCGGA
CGAGGATCTGGCGGTTTGCCACGGTGACCTGTGCCCGGACAACGTGCTGCTCGACCCTC

GTACCTGCGAGGTGACCGGGCTGATCGACGTGGGGCGGGTCGGCCGTGCGGACCGGCAC
TCCGATCTCGCGCTGGTGCTGCGCGAGCTGGCCCACGAGGAGGACCCGTGGTTCGGGCC
GGAGTGTTCCGCGGCGTTCCTGCGGGAGTACGGGCGCGGGTGGGATGGGGCGGTATCGG
AGGAAAAGCTGGCGTTTTACCGGCTGTTGGACGAGTTCTTCTGAGGGACCTGATGGTGT
TGGTGGCTGGGTAGGGTTGCGTCGCGTGGGTGACAGCACAGTGTGGACGTTGGGATCGA
TCCCCGCTCCGTGTAAATGGAGGCGCTCGTTGATCTGAGCCTTGCCCCCTGACGAACGG
CGGTGGATGGAAGATACTGCTCTCAAGTGCTGAAGCGGTAGCTTAGCTCCCCGTTTCGT
GCTGATCAGTCTTTTTCAACACGTAAAAAGCGGAGGAGTTTTGCAATTTTGTTGGTTGT
AACGATCCTCCGTTGATTTTGGCCTCTTTCTCCATGGGCGGGCTGGGCGTATTTGAAGC
GGTCGAGGGGGGGCCCGGTACC

SacI-Linker

PsaD 5′ UTR

Linker(NdeI/EcoRI)

YFP gene

HA tag (Not expressed in this construct)

Linker

PsaD 3′ UTR

Linker

hsp-rbcs2 5′

aphVIII

Rbcs2 intron

hsp-rbcs2 3′

Linker-KpnI

>HU@pNYAN-aphVIII

GAGCTCCACCGCGGTGGCGGCCGCTCTAGCTAGAACTAGTGGATCGATCCCACACACC
TGCCCGTCTGCCTGACAGGAAGTGAACGCATGTCGAGGGAGGCCTCACCAATCGTCACA
CGAGCCCTCGTCAGAAACACGTCTCCGCCACGCTCTCCCTCTCACGGCCGACCCCGCAG
CCCTTTTGCCCTTTCCTAGGCCACCGACAGGACCCAGGCGCTCTCAGCATGCCTCAACA
ACCCGTACTCGTGCCAGCGGTGCCCTTGTGCTGGTGATCGCTTGGAAGCGCATGCGAAG
ACGAAGGGGCGGAGCAGGCGGCCTGGCTGTTCGAAGGGCTCGCCGCCAGTTCGGGTGCC
TTTCTCCACGCGCGCCTCCACACCTACCGATGCGTGAAGGCAGGCAAATGCTCATGTTT
GCCCGAACTCGGAGTCCTTAAAAAGCCGCTTCTTGTCGTCGTTCCGAGACATGTTAGCA
GATCGCAGTGCCACCTTTCCTGACGCGCTCGGCCCCATATTCGGACGCAATTGTCATTT
GTAGCACAATTGGAGCAAATCTGGCGAGGCAGTAGGCTTTTAAGTTGCAAGGCGAGAGA
GCAAAGTGGGACGCGGCGTGATTATTGGTATTTACGCGACGGCCCGGCGCGTTAGCGGC
CCTTCCCCCAGGCCAGGGACGATTATGTATCAATATTGTTGCGTTCGGGCACTCGTGCG
AGGGCTCCTGCGGGCTGGGGAGGGGGATCTGGGAATTGGAGGTACGACCGAGATGGCTT
GCTCGGGGGGAGGTTTCCTCGCCGAGCAAGCCAGGGTTAGGTGTTGCGCTCTTGACTCG
TTGTGCATTCTAGGACCCCACTGCTACTCACAACAAGCCCATATGGCCCTGCTCATGC
GTTCTCAGACCCTGCGCCCCGTCTCGGCGGTGGCCTCCCGCCGCGTCTCCGTGGTCGTC
CGTGCTCAGGCTGGCGCTGAGAAAGCGCTTCGAACACCACGGGCAAGGCTAAACTTGT
GGAGGCGATCGCTACTGAGGTGGACTGACCAAGGACGTCGCTGCGAAGGCCTTTGACT
CCCTGATTGGCGGCATTGAGGACGCCCTCATCAACGGTGACCGCGTGAGTTGATCATAT
GTTTGCAGTCTTTCGCGGGGATTCAGTCGCGCCGTGAGAATTGTTCATTCCGGCAGCCA
GCATAGAACGCCGGGGTTACGAGGCGTAGTATTTGCAGTCTTAATACGCATATACATAG
CGCAGCAGTTACGCCTGGAATGGCAACTTGCGCTCGGGGGACTTTGGGGCCACGCTCGC
GCCATTTCGCACTGGCGCCTTGCCGGCGGCACCGGCACGAGCATGGGGCCGAAGATGCG
CGCGGTAGTCGTGTGTGGTTATCTTTCCGAATGGGTTTGGGCGGCTGCCGGTACGCCAA
CCCACCCTTCAAACCCACCCAGACACCGCTGAGCGACAGTCAGCAGCGCAGCCTATTAC
ACCCCCCATGTGCACAGCTTGTTGTTCGTCAGGGGTGGGGTTGCCCTTGTCGTGGACCG
AAGATAGGGCGCCAGCAGCCTGGACCTTGCACTGGTTGGTGTGTAGCTTGCCGAGCTAA
ACCCGAACCCTGGTCCTCCTTTTGGTTGCTGATGGGACCGTGTGCATTCCTGGCTCATG
GCGGTGCGCAGGTGACCATCGTGGGCTTCGGTAAGTCGCGCACGCCCTTGCCGCCGTAT
GCAAAGTACTGGGCGCACCCAGCCGCGCCGAGTCATGTTGGGGGGGCGTGCGTGGGGT
GAGGGGGATTGGACTCCACTGCACTACCGTACTCTCGCACCGAGCGCCCCGGCGATGCA

TTACAGGGCTGCAGTCACTAGCTGTGCTTTCGGGTTCTGCATACGTTCATACGCAATCT
TTATGTCACCACAGGCACGTTTGAGGTGCGCGAGCGCGCTGCGCGCCAGGGCCGCAACC
CCAGCACCGGCGCGGTCCTCCAGATCGCCGCCAGCAAGGTGGGTTGGCTCGAGGATGCA
GCAAGCTATGGGCTCATGCAACGTTGTGCTATGGGTGGTGCCTGCCGCTGCGTGCATGA
CCACGTGGGGTCGTCGGATGGGCGGACAACGCAGAGAGGGAGGTTGGCGTGGGCCAGTG
ATTGGCTCGCGGCCCATGAGGATGGGGTTGGGGTGCTGGCGGCGAGTGAGTTTGGGCCG
CCACCCGCCACCGCCGCTGCTGCTCGTGGACAGCAACAGTCGCGTTGTTGGCCTCATAC
TCCTCTCAAATCTGCCCGTCGCCGCCTGCGTGTAGGCCCCCGTGTTCAAGGCCTCAGTT
GGCCTGAAGGATGCCGTGAACGGCCGGGAGCCCAAGCCCGCGGCTGCCAAGGCCGCAGC
TGCCAAGGCCGCGGCGGCCAAGCCCGCTGCGCCGAAGCCGGCTGCGGCTAAGCCCGCGG
CTCCTCAGCCCGCGGCTCCTAAGCCCGCGGCTCCTAAGCCCGGCCCCAAGAAGGAATTC
GGCGGCTCCGGCGGCATGGTGAGCAAGGGCGAGGAGCTGTTCACCGGGGTGGTGCCCAT
CCTGGTCGAGCTGGACGGCGACGTAAACGGCCACAAGTTCAGCGTGTCCGGCGAGGGCG
AGGGCGATGCCACCTACGGCAAGCTGACCCTGAAGCTGATCTGCACCACCGGCAAGCTG
CCCGTGCCCTGGCCCACCCTCGTGACCACCCTGGGCTACGGCCTGCAGTGCTTCGCCCG
CTACCCCGACCACATGAAGCAGCACGACTTCTTCAAGTCCGCCATGCCCGAAGGCTACG
TCCAGGAGCGCACCATCTTCTTCAAGGACGACGGCAACTACAAGACCCGCGCCGAGGTG
AAGTTCGAGGGCGACACCCTGGTGAACCGCATCGAGCTGAAGGGCATCGACTTCAAGGA
GGACGGCAACATCCTGGGGCACAAGCTGGAGTACAACTACAACAGCCACAACGTCTATA
TCACCGCCGACAAGCAGAAGAACGGCATCAAGGCCAACTTCAAGATCCGCCACAACATC
GAGGACGGCGGCGTGCAGCTCGCCGACCACTACCAGCAGAACACCCCCATCGGCGACGG
CCCCGTGCTGCTGCCCGACAACCACTACCTGAGCTACCAGTCCGCCCTGAGCAAAGACC
CCAACGAGAAGCGCGATCACATGGTCCTGCTGGAGTTCGTGACCGCCGCCGGGATCACT
CTCGGCATGGACGAGCTGTACAAGTAACGATACCCCTACGACGTGCCCGACTACGCCTA
CCCCTACGACGTGCCCGACTACGCCGATCGATCCGGACCGTACCCCTACGACGTGCCCG
ACTACGCCGCTAGCAGTATCTAACTGAGTAATTCTGGCAGCAGCTGGACCGCCTGTACC
ATGGAGAAGAGCTTTACTTGCCGGGATGGCCGATTTCGCTGATTGATACGGGATCGGAG
CTCGGAGGCTTTCGCGCTAGGGGCTAGGCGAAGGGCAGTGGTGACCAGGGTCGGTGTGG
GGTCGGCCCACGGTCAATTAGCCACAGGAGGATCAGGGGGAGGTAGGCACGTCGACTTG
GTTTGCGACCCCGCAGTTTTGGCGGACGTGCTGTTGTAGATGTTAGCGTGTGCGTGAGC
CAGTGGCCAACGTGCCACACCCATTGAGAAGACCAACCAACTTACTGGCAATATCTGCC
AATGCCATACTGCATGTAATGGCCAGGCCATGTGAGAGTTTGCCGTGCCTGCGCGCGCC
CCGGGGGCGGGGGGGGGACGGGTGGGGGTAGGGGGTCTCACGGAACAGCACGCTAGG

GGTCAGGGGGGGGGGGGGGGCGCAGTTTAGCTGACCAGCCGTGGGATGATGCACGCATTT
GCAAGGACAGGGTAATCACAGCAGCAACATGGTGGGCTTAGGACAGCTGTGGGTCAGTG
GACGGACGGCAGGGGAGGGACGGCGCAGCTCGGGAGACAGGGGGAGACAGCGTGACTGT
GCAATCAAGCTTATCGATACCGTCGACCTCGACGCTGAGGCTTGACATGATTGGTGCGT
ATGTTTGTATGAAGCTACAGGACTGATTTGGCGGGCTATGAGGGCGGGGGAAGCTCTGG
AAGGGCCGCGATGGGGCGCGCGGCGTCCAGAAGGCGCCATACGGCCCGCTGGCGGCACC
CATCCGGTATAAAAGCCCGCGACCCCGAACGGTGACCTCCACTTTCAGCGACAAACGAG
CACTTATACATACGCGACTATTCTGCCGCTATACATAACCACTCAGCTAGCTTAAGATC
CCATCAAGCTTGCATGCCGGGCGCGCCAGAAGGAGCGCAGCCAAACCAGGATGATGTTT
GATGGGGTATTTGAGCACTTGCAACCCTTATCCGGAAGCCCCCTGGCCCACAAAGGCTA
GGCGCCAATGCAAGCAGTTCGCATGCAGCCCCTGGAGCGGTGCCCTCCTGATAAACCGG
CCAGGGGGCCTATGTTCTTTACTTTTTTACAAGAGAAGTCACTCAACATCTTAAAATGG
CCAGgtgagtcgacgagcaagcccggcggatcaggcagcgtgcttgcagatttgacttg
caacgcccgcattgtgtcgacgaaggcttttggctcctctgtcgctgtctcaagcagca
tctaaccctgcgtcgccgtttccatttgcagGATGGCCACTCCGCCCTCCCCGGTGCTG
AAGAATTTCGAAGCATGGACGATGCGTTGCGTGCACTGCGGGGTCGGTATCCCGGTTGT
GAGTGGGTTGTTGTGGAGGATGGGGCCTCGGGGGCTGGTGTTTATCGGCTTCGGGGTGG
TGGGCGGGAGTTGTTTGTCAAGGTGGCAGCTCTGGGGGCCGGGGTGGGCTTGTTGGGTG
AGGCTGAGCGGCTGGTGTGGTTGGCGGAGGTGGGGATTCCCGTACCTCGTGTTGTGGAG
GGTGGTGGGGACGAGAGGGTCGCCTGGTTGGTCACCGAAGCGGTTCCGGGGCGTCCGGC
CAGTGCGCGGTGGCCGCGGGAGCAGCGGCTGGACGTGGCGGTGGCGCTCGCGGGGCTCG
CTCGTTCGCTGCACGCGCTGGACTGGGAGCGGTGTCCGTTCGATCGCAGTCTCGCGGTG
ACGGTGCCGCAGGCGGCCCGTGCTGTCGCTGAAGGGAGCGTCGACTTGGAGGATCTGGA
CGAGGAGCGGAAGGGGTGGTCGGGGGAGCGGCTTCTCGCCGAGCTGGAGCGGACTCGGC
CTGCGGACGAGGATCTGGCGGTTTGCCACGGTGACCTGTGCCCGGACAACGTGCTGCTC
GACCCTCGTACCTGCGAGGTGACCGGGCTGATCGACGTGGGGCGGGTCGGCCGTGCGGA
CCGGCACTCCGATCTCGCGCTGGTGCTGCGCGAGCTGGCCCACGAGGAGGACCCGTGGT
TCGGGCCGGAGTGTTCCGCGGCGTTCCTGCGGGAGTACGGGCGCGGGTGGGATGGGGCG
GTATCGGAGGAAAAGCTGGCGTTTTACCGGCTGTTGGACGAGTTCTTCTGAGGGACCTG
ATGGTGTTGGTGGCTGGGTAGGGTTGCGTCGCGTGGGTGACAGCACAGTGTGGACGTTG
GGATCGATCCCCGCTCCGTGTAAATGGAGGCGCTCGTTGATCTGAGCCTTGCCCCCTGA
CGAACGGCGGTGGATGGAAGATACTGCTCTCAAGTGCTGAAGCGGTAGCTTAGCTCCCC
GTTTCGTGCTGATCAGTCTTTTTCAACACGTAAAAAGCGGAGGAGTTTTGCAATTTTGT

TGGTTGTAACGATCCTCCGTTGATTTTGGCCTCTTTCTCCATGGGCGGGCTGGGCGTAT TTGAAGCGGTCGAGGGGGGCCCGGTACC

SacI-Linker

PsaD 5' UTR

HLP gene (NdeI, Exon/Intron)

GS Linker

YFP gene

HA tag (Not expressed in this construct due to the stop codon in YFP)

Linker

PsaD 3' UTR

Linker

hsp-rbcs2 5'

aphVIII

Rbcs2 intron

hsp-rbcs2 3'

Linker-KpnI

This SacI-KpnI fragment was connected to SacI-KpnI sites of pBluescriptII KS+ (NCBI accession# X52327).

HU@pNYAN was constructed based on pGenD-C3HA, which was a generous gift from Dr. Ritsu Kamiya (Univ of Tokyo). The original construct pGenD PSAD was constructed by Fisher and Rochaix (Fisher and Rochaix (2001) Mol. Genet. Genomics 265, 888-894), available from Chlamydomonas genetic center.
