# Supplementary material for: Chloroplast nucleoids as a transformable network revealed by live imaging with a microfluidic device
Source: Commun Biol. 2018 May 17;1:47. doi: 10.1038/s42003-018-0055-1 (PMC6123815; doi:10.1038/s42003-018-0055-1)
Supplement: Supplementary file 2 — Description of Additional Supplementary Files [file 42003_2018_55_MOESM2_ESM.docx]

**Description of Additional Supplementary Files**

File Name: Supplementary Movie 1

Description: **Live-imaging of dividing chloroplasts and chloroplast nucleoid behaviors in *Chlamydomonas reinhardtii* cells (× approximately 2,000 speed).**

File Name: Supplementary Movie 2

Description: **Chloroplast nucleoids during cell/chloroplast division.**

Chloroplast nucleoid behaviors were monitored in a single *Chlamydomonas reinhardtii* cell expressing HU:YFP (× approximately 2,000 speed). The HU:YFP signal (yellow) and the autofluorescence from chlorophyll (red) have been merged.

File Name: Supplementary Movie 3

Description: **Emergence of *de novo* chloroplast nucleoids in the *moc* mutant.**

Chloroplast nucleoid behaviors were monitored in a single moc mutant cell (× approximately 2,000). The HU:YFP signal (yellow) and the autofluorescence from chlorophyll (red) have been merged. After the chloroplast divided, the *moc*-type chloroplast nucleoid did not diffuse, but was transmitted to only one chloroplast (left). The other chloroplast lacked any detectable chloroplast nucleoids (right). However, minute yellow signals emerged and fused, indicating a chloroplast nucleoid formed in the daughter chloroplast (right).

File Name: Supplementary Movie 4

Description: **Rapid development of new chloroplast nucleoids in the *moc* mutant.** Chloroplast nucleoid behaviors were monitored in a single *moc* mutant cell (× approximately 2,400). The HU:YFP signal (yellow) and the autofluorescence from chlorophyll (red) have been merged. After the chloroplast divided, the moc-type chloroplast nucleoid was transmitted to only one chloroplast (left). Minute chloroplast nucleoid particles emerged and fused together to establish normal-sized nucleoids in the other chloroplast.

File Name: Supplementary Movie 5

Description: **Particle-tracking analysis to visualize the growth of a new chloroplast nucleoid.**

The intensity and distribution of the HU:YFP signal underwent a surface plot analysis based on Supplemental Movie 5 (× approximately 2,400).
